# Rhomboid intramembrane protease RHBDL4 triggers ER-export and non-canonical secretion of membrane-anchored TGFα

**DOI:** 10.1038/srep27342

**Published:** 2016-06-06

**Authors:** Lina Wunderle, Julia D. Knopf, Nathalie Kühnle, Aymeric Morlé, Beate Hehn, Colin Adrain, Kvido Strisovsky, Matthew Freeman, Marius K. Lemberg

**Affiliations:** 1 Zentrum für Molekulare Biologie der Universität Heidelberg (ZMBH), DKFZ-ZMBH Allianz, Im Neuenheimer Feld 282, 69120 Heidelberg, Germany; 2 Institute of Organic Chemistry and Biochemistry, Academy of Sciences of the Czech Republic, Prague, Czech Republic; 3 Instituto Gulbenkian de Ciência, Rua da Quinta Grande, 6, 2780-156, Oeiras, Portugal; 4 Sir William Dunn School of Pathology, University of Oxford, South Parks Road, Oxford OX1 3RE, UK

## Abstract

Rhomboid intramembrane proteases are the enzymes that release active epidermal growth factor receptor (EGFR) ligands in *Drosophila* and *C. elegans*, but little is known about their functions in mammals. Here we show that the mammalian rhomboid protease RHBDL4 (also known as Rhbdd1) promotes trafficking of several membrane proteins, including the EGFR ligand TGFα, from the endoplasmic reticulum (ER) to the Golgi apparatus, thereby triggering their secretion by extracellular microvesicles. Our data also demonstrate that RHBDL4-dependent trafficking control is regulated by G-protein coupled receptors, suggesting a role for this rhomboid protease in pathological conditions, including EGFR signaling. We propose that RHBDL4 reorganizes trafficking events within the early secretory pathway in response to GPCR signaling. Our work identifies RHBDL4 as a rheostat that tunes secretion dynamics and abundance of specific membrane protein cargoes.

Intramembrane proteases perform a wide range of important functions ranging from activation of membrane-tethered molecules to regulated protein degradation^1^. Rhomboids are intramembrane serine proteases^2^, first described in *Drosophila*, where Rhomboid-1 was initially defined as the key regulator of epidermal growth factor receptor (EGFR) signaling^3^,^4^. Heterologous expression of *Drosophila* Rhomboid-1 in mammalian tissue culture cells illuminated its function in the release of growth factors. Rhomboid-1 cleaves the EGFR ligand Spitz in its transmembrane (TM) domain, thereby inducing secretion of the bioactive ligand and signaling to neighboring cells^5^. In addition to transcriptional control of *rhomboid-1*^6^, this release pathway is mediated by regulated substrate trafficking. The transport factor Star guides Spitz from the endoplasmic reticulum (ER), thereby bringing it into contact with the Golgi-localized Rhomboid-1 protease^5^. A related function has been reported in *C. elegans for* the rhomboid protease ROM-1 in processing of the EGFR ligand LIN-3L^7^.

Despite widespread conservation of signaling pathways, this is a case where mammals use a different mechanism: EGFR ligand processing in mammals is mainly driven by sheddases of the ADAM-type (‘a disintegrin and metalloprotease’)^8^, and EGFR ligand processing by rhomboids may have a modulatory effect only^9^. Transforming growth factor α (TGFα), the best-characterized mammalian EGFR ligand, is activated by ADAM17, also known as TACE (‘TNFα converting enzyme’)^10^. Membrane-anchored TGFα moves to the plasma membrane accompanied by PDZ domain proteins, where ADAM17 cleaves it just outside its TM domain, releasing the active EGFR ligand^11^. Nonetheless, several reports indicate that processing and maturation of TGFα is more complex, including regulation by G-protein coupled receptor (GPCR) signaling^12^,^13^,^14^. Furthermore, in some contexts release of TGFα has been shown to be sensitive to serine protease inhibitors^15^. The rhomboid intramembrane protease RHBDL4 (also known by its gene name *Rhbdd1*) has been recently suggested to be an alternative shedding enzyme for TGFα at the plasma membrane^16^. This contrasts with an earlier report that showed that RHBDL4 localizes to the ER, where it functionally interacts with the ER-associated protein degradation (ERAD) pathway^17^. RHBDL4, which is phylogenetically distinct from the three other rhomboid proteases in the mammalian secretory pathway^18^, has also been linked to other processes such as regulating apoptosis via c-Jun and exosome secretion^19^,^20^. Overall, there is no clear consensus about the function in mammalian cells of RHBDL4.

In this study we set out to address the apparent discrepancy between the function of RHBDL4 in the ER and the protease’s putative role in TGFα biology. We find that RHBDL4 enhances ER-to-Golgi trafficking of the unprocessed, full-length TGFα but, contrary to a previous report^16^, does not directly contribute to its shedding. Unexpectedly, RHBDL4 regulates secretion of TGFα, and other cargoes, in extracellular microvesicles. This process alters the balance between the canonical shedding pathway and an alternative pathway of microvesicle release of membrane tethered, inactive TGFα. Our data also reveal that RHBDL4 can be regulated by GPCR signaling, a process associated with EGFR transactivation. These observations suggest that RHBDL4 is a nexus integrating intercellular signals via GPCRs, tuning the signaling output by modulating secretory cargo trafficking.

## Results

### RHBDL4 can promote release of full-length proTGFα

**Figure 1:**
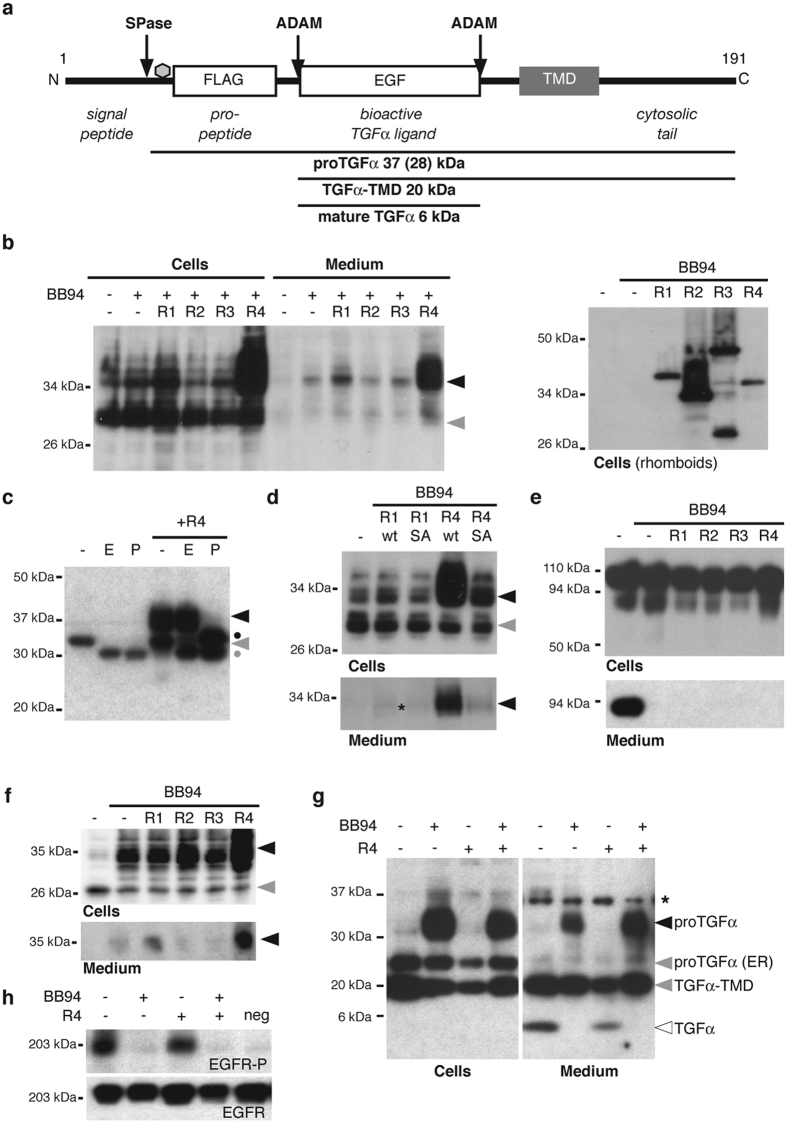
RHBDL4 triggers secretion of proTGFα in an ADAM protease-independent manner. (**a**) Schematic representation of pre-proTGFα. Position of the FLAG-tag used in this study is indicated. SPase, signal peptidase; grey hexagon, N-linked glycosylation site; TMD, TM domain. (**b**) Ectopically expressed RHBDL4 in Hek293T cells (and to a lower extent also RHBDL1) trigger generation of a 37-kDa form of proTGFα-FLAG (black arrow) that is secreted into the tissue culture supernatant. In contrast, the 28-kDa form lacking complex-type glycans in the pro-peptide is not significantly secreted (grey arrow). Secreted proTGFα-FLAG was detected at low level in absence of rhomboid overexpression, so that an overexposed blot is shown. Expression of ectopically expressed RHBDLs was detected by the HA-tag (right panel). The assay, except lane 1/7, was performed in the presence of 10 μM BB94 to inhibit shedding by ADAM proteases. (**c**) EndoH and PNGaseF treatment of cell extracts shows that the 37-kDa form of proTGFα-FLAG (black arrow) is EndoH-insensitive demonstrating that it is localized to the late secretory pathway. In contrast, the 28-kDa species representing the ER form (grey arrow) is EndoH-sensitive. Dot, deglycosylated form. (**d**) Release of proTGFα-FLAG is specifically caused by rhomboid activity since the active site mutant of RHBDL1 (S247A) and RHBDL4 (S144A) show no significant effect. (**e**) Effect is specific to proTGFα-FLAG since FLAG-tagged S1P, which is cleaved by BB94-sensitive sheddase, is not released upon RHBDL4 overexpression. (**f**) Assay as in (**b**) using HCT116 cells. (**g**) Assay as in (**b**) using untagged proTGFα. Western blotting using a TGFα-specific antibody reveals in addition to higher MW form (black arrow) secretion of 20-kDa species lacking the pro-peptide (TGFα-TMD) and mature 6-kDa TGFα. ProTGFα lacking complex type glycans (ER form) is not secreted. (**h**) Incubation of conditioned media from cell-based assay as in (**g**) with medium starved A431 cells shows that higher MW forms cannot efficiently activate the EGFR whereas mature TGFα observed in absence of BB94 leads to robust signaling. EGFR-P, phosphorylated EGFR; neg, A431 cells treated with fresh medium.

There is evidence for an ADAM17-independent secretion mechanism for TGFα, including activity sensitive to serine protease inhibitor 3,4-dichloroisocoumarin (DCI)^15^,^21^ and a recently suggested link to RHBDL4^16^. TGFα maturation is complex: prior to shedding of the active growth factor from the plasma membrane, TGFα also incurs cleavage of the N-terminal pro-domain, and a variety of modifications including N-linked glycosylation (Fig. 1a)^11^. We therefore set out to evaluate the impact of mammalian rhomboid proteases on TGFα biology in cell-based assays. We used the metalloprotease inhibitor BB94 to block both pro-peptide cleavage and shedding by ADAM proteases, and co-expressed FLAG-tagged proTGFα^16^ with the four known mammalian secretory pathway rhomboid proteases in Hek293T cells (Fig. 1b). Surprisingly, RHBDL4 triggered extensive BB94-insensitive release of a 37-kDa form of proTGFα into the media, suggesting a secretion mechanism for full-length proTGFα, distinct from canonical shedding of the 6-kDa extracellular active ligand by ADAM proteases (see Fig. 1g). Shedding of a full-length 37-kDa form was broadly specific to RHBDL4 (although RHBDL1, a Golgi enzyme, had a very slight similar effect) (Fig. 1b). Traces of a faster migrating species, which runs slightly above the 28-kDa species found in the cell extracts, were also secreted. The unexpected high molecular weight of both secreted forms implies that proTGFα is not shed in a classical manner by the rhomboid proteases but instead is released as full-length protein (see below).

Using the deglycosylating enzymes EndoH and PNGaseF to distinguish ER- from Golgi-modified glycans in the cell extracts, we found that the 37-kDa form of proTGFα has been modified in the Golgi whereas the 28-kDa form represents the ER-resident species harboring only the core glycan structure (Fig. 1a,c). This result indicates that the secreted 37-kDa species, whose generation is enhanced by RHBDL4, is proTGFα that has passed through the Golgi apparatus.

Secretion of the 37-kDa TGFα species in cell extracts and media fraction was dependent on the active site serine of RHBDL4 (Fig. 1d), implying a requirement for the proteolytic activity of RHBDL4 to induce this non-canonical proTGFα secretion. In contrast, RHBDL4 did not affect S1P (site 1-protease), a type I membrane protein that upon overexpression is secreted by a BB94-sensitive shedding event (Fig. 1e), indicating that the effect is specific. Identical BB94-insensitive release of a higher molecular weight form of proTGFα was observed in HCT116, HeLa and COS7 cells (Fig. 1f and data not shown), demonstrating a mechanism general to a range of cell types. Overall, these results identify a previously unrecognized secretion mechanism for full-length proTGFα.

To test the relationship of this non-canonical secretion mechanism to EGFR signaling, we co-expressed untagged proTGFα with RHBDL4 and observed that RHBDL4 augmented the release of the higher MW species while reducing the amount of the 6-kDa secreted species. This is consistent with a role for RHBDL4 in remodeling the secretory fate of cargo proteins (Fig. 1g).

We next examined the activation of the EGFR by conditioned tissue culture supernatants (Fig. 1h). By using an antibody against the EGF domain of TGFα we also observed generation of a membrane-tethered 20-kDa TGFα species lacking the pro-peptide (subsequently described as TGFα-TMD) that is also secreted into the tissue culture supernatant (Fig. 1a,g and below). Neither secreted higher molecular weight form of TGFα activated the EGFR on medium starved A431 cells (Fig. 1h). Contrary to a previous report^16^, this indicated that TGFα released by RHBDL4 (in presence of BB94 to prevent canonical shedding) is not bioactive. In contrast to the RHBDL4-induced release of TGFα, when ADAM proteases were not inhibited, significantly less higher-molecular-weight proTGFα forms were detected, both in the cell extract and in the media fraction, and trimming to the 6-kDa mature growth factor was observed (Fig. 1a,g). This form efficiently activated the EGFR in A431 cells (Fig. 1h). Taken together, these results reveal an unexpected link of a rhomboid protease to trafficking and secretion of the unprocessed, full-length pro-form of an EGFR ligand.

### RHBDL4 localizes to the ER in multiple cell lines

**Figure 2:**
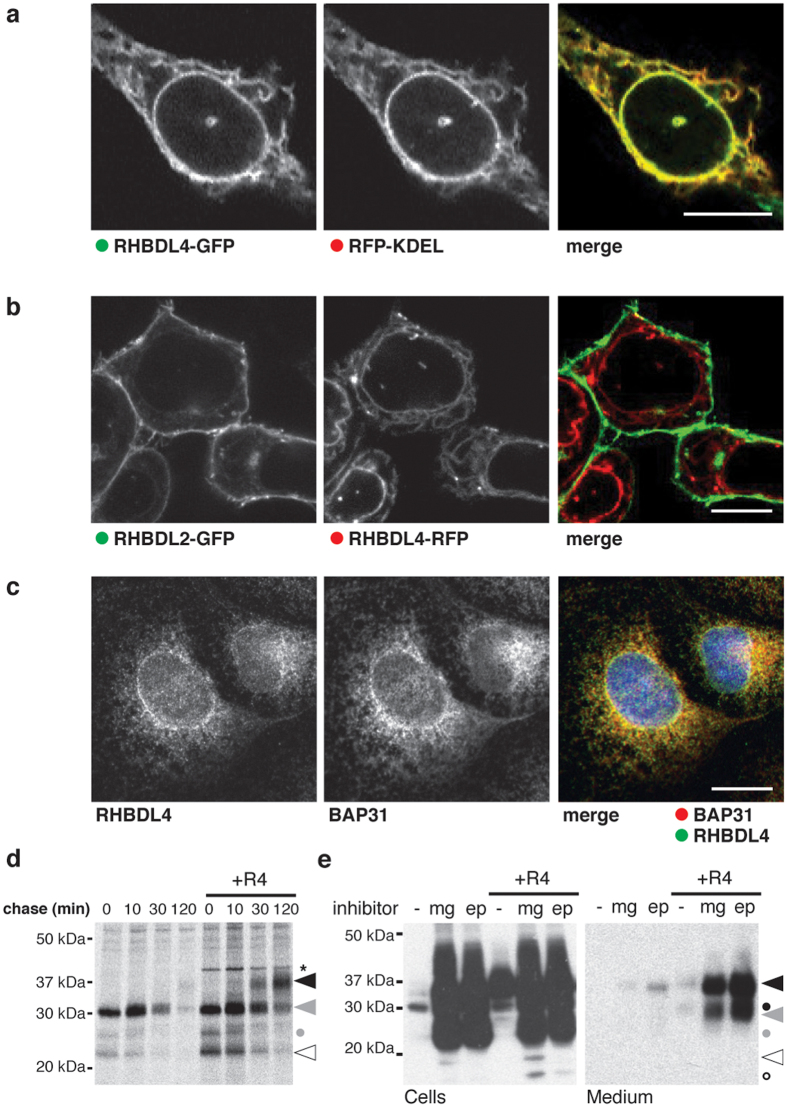
RHBDL4 localizes to ER and rescues proTGFα from ERAD. (**a**) RHBDL4-GFP co-localizes to the ER marker RFP-KDEL in HCT116 cells. Scale bar, 5 μM. (**b**) Fluorescent micrograph of HCT116 cells co-expressing RHBDL2-GFP and RHBDL4-RFP. Scale bar, 5 μM. (**c**) Immunofluorescence analysis of untransfected COS7 cells shows RHBDL4 co-localization with the ER protein BAP31. Scale bar, 5 μM. (**d**) Metabolic pulse label and chase experiment shows that the 37-kDa proTGFα species is generated with a lag phase of 30 minutes, whereas lower molecular weight degradation intermediates (white arrow and grey dot) appear directly after the pulse and chase away over time. Asterisk, unspecific band. (**e**) Treatment of Hek293T cells ectopically expressing proTGFα-FLAG with the proteasome inhibitor MG132 (mg; 5 μM) and epoxomicin (ep; 2 μM) leads to massive increase of the 37-kDa species (black arrow) and to various unglycosylated forms (circles) indicating that it is degraded to a high extent by the ERAD pathway. Whereas the 37-kDa form is secreted, mimicking the effect caused by RHBDL4 overexpression, the lower molecular weight species are only detected in the cell lysates (grey and open symbols).

Next we asked, where in the secretory pathway RHBDL4 acts. The subcellular localization of RHBDL4 has been controversial. Although we previously observed that in Hek293T cells RHBDL4 localizes to the ER^17^, Wang and colleagues recently described plasma membrane localization of RHBDL4 in HCT116 cells by immunofluorescence analysis^16^. Since we have seen that formaldehyde fixation can impair immunofluorescence analysis of RHBDL4 (result not shown), we used living cells to assess RHBDL4 subcellular localization. We imaged HCT116 cells co-transfected with RHBDL4-GFP and the ER marker RFP-KDEL and detected complete co-localization (Fig. 2a). Consistent with this, we did not detect any overlap of RHBDL4-RFP with the plasma membrane-localized RHBDL2-GFP (Fig. 2b). Localization of RHBDL4 to the ER was further corroborated by immunofluorescence analysis of endogenous RHBDL4 in acetone fixed COS7 cells (Fig. 2c). Although these results formally do not rule out that a subfraction of RHBDL4 could be elsewhere in some contexts, we conclude that the predominant localization of endogenous and ectopically expressed RHBDL4 is the early secretory pathway.

### RHBDL4 redirects proTGFα from proteasomal degradation towards secretion

To find out whether RHBDL4-induced increase of intracellular proTGFα is caused by increased biosynthesis rate or decreased degradation, we performed a pulse-chase analysis. Directly after a pulse label in cells transfected with FLAG-tagged TGFα, the 28-kDa ER pro form and traces of a lower molecular weight species were detected (Fig. 2d). With a half-life shorter than 20 minutes this species decreased and only traces of the 37-kDa mature species were generated. Since the experiment was performed in presence of BB94, blocking ADAM-mediated maturation and shedding, we conclude that proTGFα is degraded. In contrast, in cells co-expressing RHBDL4, the 28-kDa form almost quantitatively converted to the 37-kDa species, indicating that the effect is caused by increased ER export and subsequent addition of Golgi modifications. Moreover, slightly more of a 22- and 26-kDa species were detected. However, in contrast to the 37-kDa form that is generated with a lag phase of 30 minutes and increases at 120 minutes, the lower molecular weight species were already present directly after the pulse and rapidly chased away, suggesting that they represent degradation intermediates caused by defective synthesis. Consistent with a previous report^22^ we observed, using proteasome inhibitors, that tagged proTGFα is highly susceptible to proteasomal degradation (Fig. 2e). This demonstrates that under steady state conditions the majority of FLAG-tagged proTGFα does not leave the ER but is degraded by the ERAD pathway. However, ectopic expression of RHBDL4 or inhibition of the proteasome changes this fate, increasing ER export, and resulting in secretion of the higher molecular weight form (Fig. 2e). This indicates that the majority of proTGFα is properly folded and has the potential to pass the ER quality control machinery, implying that this is a case of ERAD being used as a signal regulator rather than a quality control process. This adds to a growing number of cases of this concept^23^,^24^,^25^,^26^. Expression of RHBDL4 in presence of proteasome inhibitors increased secretion even further (Fig. 2e), indicating that turnover of proTGFα by the ERAD pathway and the RHBDL4-induced ER export are based on two independent mechanisms.

### ER export and non-canonical secretion of proTGFα are mediated by its cytoplasmic PDZ-binding domain

**Figure 3:**
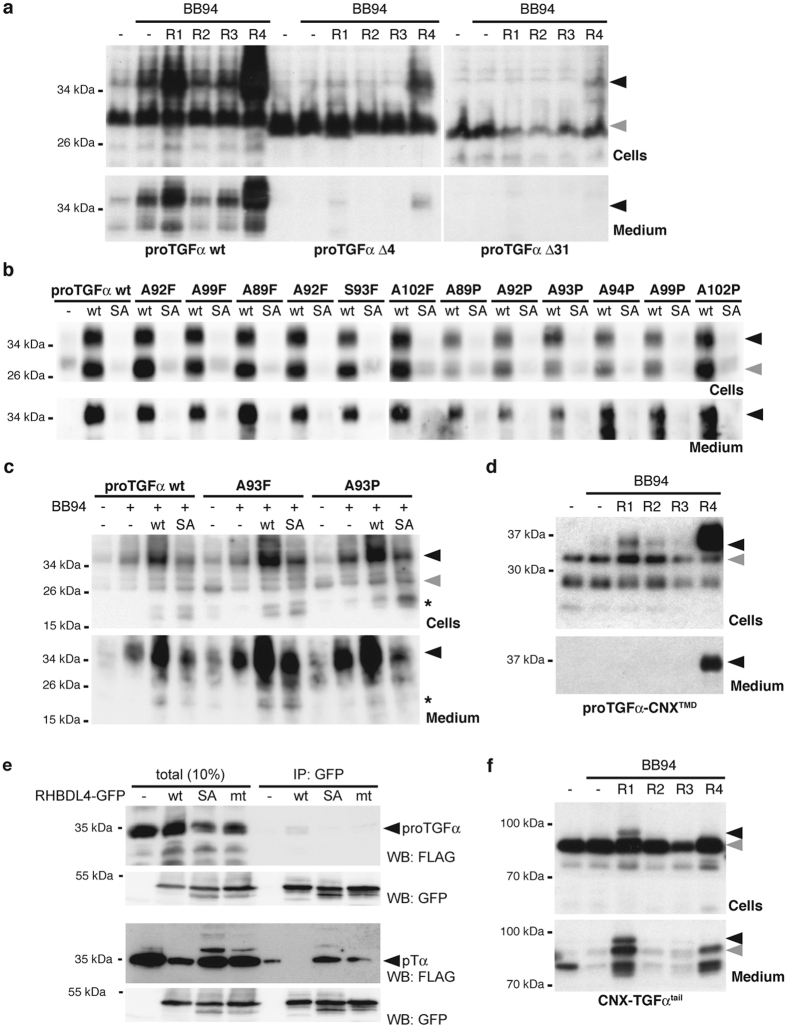
Non-canonical proTGFα secretion is mediated by its cytoplasmic tail and not the juxtamembrane region. (**a**) Deletion of carboxy-terminal PDZ-binding domain (Δ4) reduces secretion of proTGFα, whereas deletion of the entire cytoplasmic tail (Δ31) completely prevents its release. (**b**) Secretion of proTGFα triggered by RHBDL4 ectopically expressed in Hek293T cells is unaffected by mutations of putative rhomboid-like cleavage sites in the juxtamembrane and adjacent TM region of FLAG-proTGFα. (**c**) Mutation of A93F and A93P do not prevent RHBDL4-induced secretion of proTGFα and secretion of a 17-kDa proTGFα fragment (indicated by asterisk) is independent of RHBDL4 activity in HCT116 cells. (**d**) RHBDL4-triggered non-canonical proTGFα secretion does not depend on membrane integral features shown by expression of a chimeric molecule harboring the TM domain of the stable ER protein calnexin (proTGFα-CNX^TMD^). (**e**) The active site S144A mutant of RHBDL4-GFP (SA) co-immunoprecipitates the ERAD substrate pTα but not proTGFα. Mutation of L274A and L278A (mt) in the conserved ubiquitin interacting motif of RHBDL4 reduce binding of pTα as has been observed previously^17^. IP, immunoprecipitation; WB, western blot. (**f**) Carboxy-terminal tail of proTGFα is sufficient for non-canonical secretion as demonstrated by the chimeric molecule CNX-TGFα^tail^.

ER export and subsequent trafficking of proTGFα has previously been shown to be a regulated process, with various cytoplasmic regulatory factors including PDZ-domain proteins recognizing its C-terminal tail and thereby controlling its trafficking^11^. To test the role of these trafficking signals, we generated truncated proTGFα constructs lacking either the PDZ-binding domain (Δ4) or the entire cytoplasmic tail (Δ31) and compared their fate to the full-length protein (Fig. 3a). Consistent with previous reports^27^,^28^, almost no higher molecular weight species were detected for either mutant, even when pro-peptide cleavage was blocked by BB94, indicating that they do not efficiently leave the ER. Co-expression of RHBDL4 only marginally promoted trafficking of the deletion mutants, judged by the presence of higher molecular weight species. Whereas traces of proTGFαΔ4 were detected in the media fraction, no release of proTGFαΔ31 was observed. These results indicate that the cytoplasmic tail and the PDZ-binding domain of proTGFα are needed for RHBDL4 to promote trafficking and secretion.

Consistent with the release of proTGFα not being caused by RHBDL4-catalysed canonical shedding, mutation of all small and unbranched amino acids in the juxtamembrane region of proTGFα, which represent potential rhomboid recognition and cleavage sites^29^, had no significant effect on RHBDL4-induced maturation and secretion (Fig. 3b). Recently Song *et al*. suggested that in HCT116 cells RHBDL4 cleaves proTGFα at Ala-93 in the juxtamembrane region^16^. To examine this idea, the A93F and A93P mutants, which are predicted to block any rhomboid-mediated proteolysis at this putative site^29^, were expressed in presence of RHBDL4. As shown in Fig. 3c, using HCT116 cells, these mutants had no effect on secretion of the 37-kDa proTGFα form and several lower molecular weight species. Importantly, we also detected traces of a 17-kDa species that Song *et al*. referred to as RHBDL4-generated cleavage fragment^16^ in the media fraction. However, in contrast to this previous report, we observed that the presence of this species was not affected by rhomboid proteolysis, as equal amounts of it were generated in the presence of catalytically inactive RHBDL4 S144A. Moreover, none of the putative cleavage site mutants affected production of this species.

These findings support our conclusion that the RHBDL4-mediated release of TGFα is not caused by rhomboid-mediated shedding of the extracellular domain. Our finding that TGFα is not a RHBDL4 substrate is further supported by domain swap experiments with the stable ER protein calnexin, which we previously showed to resist cleavage by ectopically expressed RHBDL4^17^. Replacement of the proTGFα TM domain by the calnexin TM domain did not affect RHBDL4 induced maturation and secretion (Fig. 3d). Furthermore, we did not co-purify any proTGFα with the catalytic S144A mutant of RHDBL4, which we previously showed to act as a substrate trap for the unstable orphan α-subunit of the pre-T cell receptor (pTα) (Fig. 3e)^17^. Taken together, these results strongly support the conclusion that RHBDL4 does not cleave proTGFα but instead modulates its intracellular trafficking and subsequent release by an unknown mechanism, albeit one that is dependent on RHBDL4 protease activity. Consistent with this hypothesis, fusing the ER protein calnexin to the cytoplasmic tail of proTGFα is sufficient to allocate calnexin to the RHBDL4 (and RHBDL1) dependent secretion mechanism (Fig. 3f).

### RHBDL4 enhances ER export of a wide range of membrane proteins

**Figure 4:**
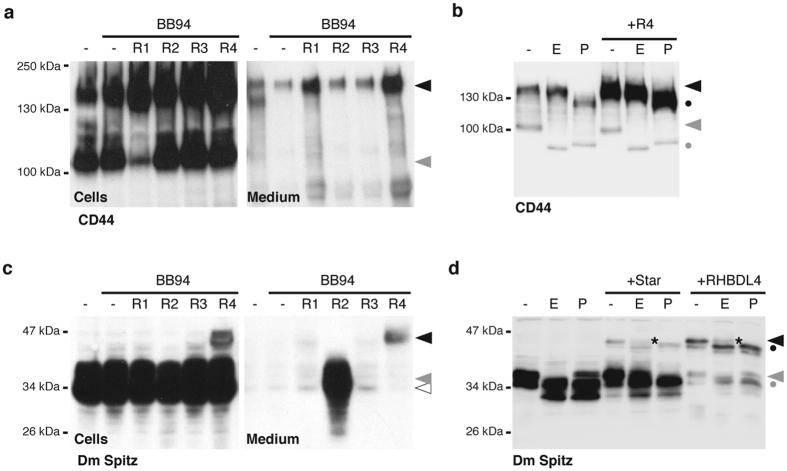
RHBDL4 enhances ER-to-Golgi trafficking of various type I membrane proteins. (**a**) RHBDL4-induced secretion of higher MW form is also observed for ectopically expressed CD44 (to a lower extend also by RHBDL1). (**b**) EndoH and PNGaseF treatment of cell extracts shows that higher molecular weight forms of CD44 (black arrow) localize to the late secretory pathway, whereas the lower molecular weight form (grey arrow) is ER-resident. Dot, deglycosylated form. (**c**) Cell-based rhomboid assay as in (**a**) using *Drosophila* Spitz. (**d**) RHBDL4-induced shift of Spitz to a higher molecular weight form mimics Star-mediated ER-to-Golgi transport. Asterisk, EndoH-insensitive fraction of Spitz.

Next we asked whether the RHBDL4-mediated effect was specific to the trafficking of proTGFα, or if the effect is more widespread. The cytoplasmic tail of the type I membrane protein CD44, which is a cell adhesion factor that is expressed in a broad range of tissues, has similarly been implicated in its cellular trafficking^30^. Upon expression of FLAG-tagged CD44, we detected a BB94-independent release of a 150-kDa species into the tissue culture supernatant (Fig. 4a). As observed for proTGFα, coexpression of CD44 with RHBDL4 increased the level of the late secretory pathway form and its subsequent release into the media fraction, whereas the 100-kDa ER-resident species was not significantly affected (Fig. 4a,b). This implies that the ability of RHBDL4 to promote secretory trafficking is not restricted to proTGFα.

To challenge this idea, we assessed the effect of RHBDL4 on the *Drosophila* Rhomboid-1 substrate Spitz, which is the closest *Drosophila* homologue of proTGFα. Spitz has been previously reported to be a substrate of RHBDL2, another mammalian rhomboid protease; upon shedding and addition of complex type glycans in the Golgi, RHBDL2 sheds a 35-kDa form of Spitz (Fig. 4c)^5^,^24^. In contrast to the conventional shedding of Spitz induced by RHBDL2, co-expression of RHBDL4 led to the generation of a 47-kDa form in both cell extract and the media fraction. This higher molecular weight form contains EndoH-insensitive glycans, which implies its passage through the late secretory pathway. Interestingly, a virtually identical high-molecular-weight form of Spitz is generated also upon its coexpression with the *Drosophila* transport factor Star (Fig. 4d), consistent with the effect being caused by potentiating ER-to-Golgi transport.

### proTGFα and CD44 are secreted by microvesicles

**Figure 5:**
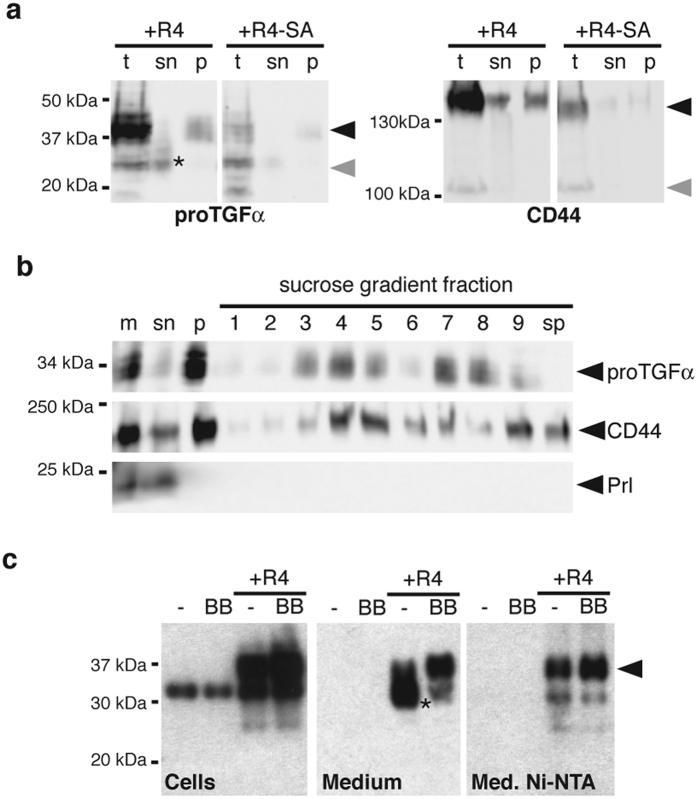
RHBDL4-induced stabilization of higher molecular weight forms leads to their detection in secreted microvesicles. (**a**) proTGFα-FLAG or CD44-FLAG were co-expressed in Hek293T cells with RHBDL4 (R4) or the catalytically inactive RHBDL4 mutant (R4-SA) in presence of 10 μM BB94. After removing cell debris, conditioned media were subjected to ultracentrifugation. The 37-kDa form of proTGFα-FLAG and a subpopulation of CD44-FLAG were recovered in the 175,000 × g pellet fraction (p), whereas the trimmed FLAG-tagged TGFα pro-peptide (indicated by asterisk) was in the supernatant fraction (sn). t, total cell extracts. (**b**) proTGFα and CD44 from the 175,000 × g pellet fraction as obtained in (**a**) float in a sucrose gradient indicating that they are secreted by microvesicles. Soluble secreted prolactin serves as negative control as it is found in the supernatant (sn) fraction only. Sucrose density ranges from 1.06 g/l for the top (fraction 1) to 1.25 g/l (fraction 9); sp, sucrose gradient pellet. (**c**) Released proTGFα-FLAG (black arrow) is trapped by a His-tag that has been introduced between TACE-cleavage site and the juxtamembrane region. In contrast, FLAG-tagged pro-peptide (asterisk) is not bound.

As well as conventional secretion of soluble ligands, membrane proteins can be released from cells in microvesicles or exosomes, which are cytoplasm-filled vesicles derived from multivesicular bodies or the plasma membrane^31^. It has been reported previously that CD44 and certain EGFR ligands can be secreted in exosomes in a signaling competent form^32^,^33^. To analyze whether the RHBDL4-dependent 37-kDa proTGFα and 150-kDa CD44 species are found in such extracellular microvesicles, the 175 000 g pellet of conditioned media from Hek293T cells co-expressing RHBDL4 and the respective ligand (Fig. 5a) were analyzed by sucrose flotation (Fig. 5b) which allows density-dependent separation of different types of vesicles, while protein aggregates are found in the pellet fraction^34^. We recovered higher molecular weight forms of both proTGFα and CD44 in fractions from the sucrose gradient (Fig. 5b) that indicated that they are associated with membrane-enclosed microvesicles. The secreted hormone prolactin was secreted in its soluble form and was not present in any vesicle-containing fraction (Fig. 5b), proving the effectiveness of the separation. We conclude that RHBDL4 promotes the release of proTGFα and CD44 in membrane-enclosed extracellular vesicles.

Upon secretion of microvesicles, type I membrane proteins such as proTGFα are orientated with their ectodomain facing the extracellular space and their C-terminus pointing towards the cytoplasm-filled inside^31^. Consistent with this topology, proTGFα with a His-tag between the juxtamembrane ADAM17 cleavage site and the TM domain was recovered from the media by Ni-NTA resin (Fig. 5c), which recognizes the His-tag. In contrast, the slightly smaller form observed in absence of BB94 was not bound by Ni-NTA, confirming that it represents the soluble ectodomain released by canonical ADAM-catalyzed shedding. These results further support the conclusion that RHBDL4-dependent ER to Golgi trafficking of full-length proTGFα and CD44 promotes their release in microvesicles.

### RHBDL4-induced ER-to-Golgi trafficking is signal regulated

Biosynthesis and trafficking of membrane proteins are regulated by a variety of signals^35^,^36^ including growth-promoting GPCR pathways^37^. For example, in a process known as transactivation, GPRC activation leads to physiological and pathological stimulation of EGFRs by a secondary, autocrine release of their ligands (see ref. 38 for review). Release of EGFR ligands is also triggered by agents that stimulate downstream targets of GPCR signaling, including the phorbol ester phorbol 12-myristate 13-acetate (PMA)^15^. The intracellular pathways that activate release of EGFR ligands during transactivation are not fully understood but, interestingly, treatment of mice by angiotensin (which activates a GPCR) leads to the generation of a 37-kDa form of TGFα *in vivo*^14^. Although this process so far has been seen in the light of ADAM-catalyzed shedding, the similarity to the RHBDL4 generated form of proTGFα prompted us to investigate whether transactivation might be linked to RHBDL4.

**Figure 6:**
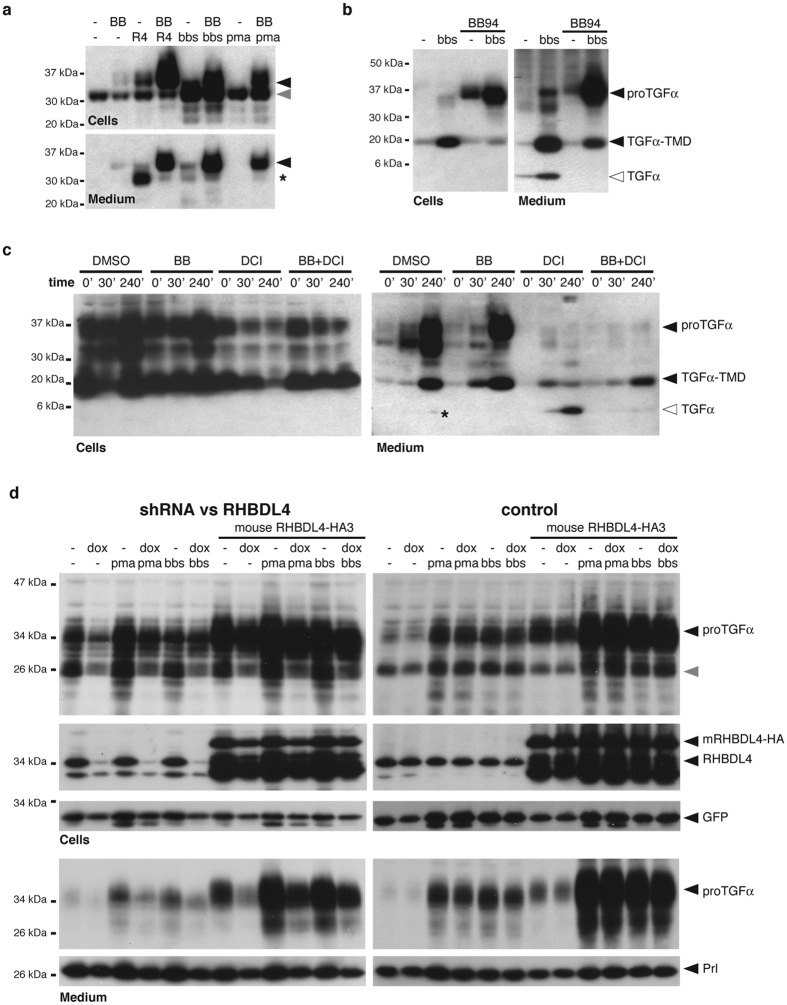
RHBDL4-mediated proTGFα-secretion boost is enhanced by transactivation. (**a**) Exosome secretion of proTGFα-FLAG in COS7 cells ectopically expressing GRPR is stimulated by PMA or bombesin (bbs), mimicking the effect caused by RHBDL4 overexpression. In absence of BB94 (BB), transactivation by PMA and bbs also affects the extent of pro-peptide cleavage subsequently reducing the amount of secreted pro-peptide (asterisk) in the media fraction. (**b**) Upon transactivation of COS7 cells with bbs, the secreted 37-kDa form of proTGFα-FLAG was processed to the 6-kDa mature form via a number of intermediates. Of note, due to a low expression the ER-resident 28-kDa form of proTGFα-FLAG was not detected (cf. Fig. 1a,g). (**c**) Time course of TGFα secretion after PMA stimulation of Hek293T ectopically expressing proTGFα-FLAG was performed in presence of BB94 (BB, 20 μM), DCI (100 μM) or both. (**d**) PMA and bbs-induced proTGFα-FLAG secretion boost can be suppressed by RHBDL4 knockdown in Hek293 T-REx cells expressing an inducible shRNA but not in control cells. This effect is rescued by ectopically expressed mouse RHBDL4 (mRHBDL4-HA). Cytosolic GFP and ectopically expressed prolactin (Prl) serve as transfection and loading controls.

The peptide hormone bombesin activates the gastrin-releasing peptide receptor (GRPR)^39^. Treatment of COS7 cells expressing the GRPR with bombesin enhanced the BB94-insensitive release of the 37-kDa form of proTGFα(Fig. 6a). Similar BB94-insensitive release of full-length proTGFα was induced by PMA (Fig. 6a). All these forms were indistinguishable from the 37-kDa form generated by RHBDL4 overexpression (Fig. 6a). Although previous studies of EGFR transactivation have shown it to be BB94-sensitive, these have primarily assayed TGFα secretion by assaying EGFR activation^12^,^13^,^14^, which would not detect changes in microvesicle secretion of proTGFα, which is unable to stimulate the EGFR (see Fig. 1h). When ADAMs were not inhibited by BB94, the 37-kDa form of proTGFα disappeared, in concert with an increase in the 20-kDa membrane-anchored form (lacking the propeptide), and the appearance of the 6-kDa soluble, bioactive ligand in the media fraction (Fig. 6b). We interpret this to mean that RHBDL4 can trigger microvesicle secretion of membrane tethered TGFα even when competing with metalloproteases that remove the prodomain and produce the bioactive soluble ligand. Importantly, in the absence of BB94, along with robust secretion of the 20-kDa species, traces of the 37-kDa species were also observed in the media fraction, indicating that trafficking into microvesicles is not an artifact of metalloprotease inhibition.

A central prediction of our model is that the observed release of the 37-kDa form would be inhibited by the serine protease inhibitor DCI, a rhomboid inhibitor^40^. This experiment is difficult, because robust release of proTGFα is detectable only several hours after stimulation (Fig. 6c), but DCI is toxic to cells over a similar time course. To help the cells survive, we expressed the antiapoptotic protein Bcl-XL^41^. As expected, DCI had a strong and specific inhibitory effect on the release of the 37-kDa form of proTGFα in response to PMA (Fig. 6c), suggesting that the effect of GPCR activation is dependent on RHBDL4 protease activity. Inhibition of ADAM proteases by BB94 blocked generation of the 6-kDa mature form, while both the 37-kDa and 20-kDa membrane-anchored forms were released by the microvesicle pathway (cf. Fig. 1a,g). Double treatment of cells with DCI and BB94, however, leads only to release of the 20-kDa form, indicating that it originates from the constitutive trafficking of TGFα that gets liberated from the pro-peptide even in presence of BB94 (but needs induced ADAM17 activity to be converted into the mature, soluble EGFR ligand^22^). Collectively, these results imply that GPCR signaling tunes ER exit of proTGFα in an RHBDL4-dependent manner, whereas microvesicle release of cargo that has reached the late secretory pathway is constitutive.

Consistent with a link between RHBDL4 and GPCR signaling, knockdown of RHBLD4 in Hek293 T-REx cells stably expressing a *RHBDL4*-specific shRNA^17^ reduced both PMA- and bombesin-induced release of proTGFα containing microvesicles, whereas no effect was observed in control cells lacking the shRNA (Fig. 6d). Ectopic expression of mouse RHBDL4 increased generation and release of the 37-kDa proTGFα form and rescued the knockdown. In contrast, secretion of the hormone prolactin was unaffected by manipulating RHBDL4 levels, demonstrating that the effect is specific and does not affect the constitutive secretory pathway.

In summary, our results show that in response to GPCR activation, RHBDL4 augments ER export of specific membrane protein cargo, thereby tuning the availability of important bioactive molecules including proTGFα. The underlying molecular mechanism, and the extent to which RHBDL4 contributes *in vivo* to EGFR transactivation and other GPCR signaling outcomes, are now important questions.

## Discussion

Ectodomain shedding is a universal mechanism that converts inactive TM proteins into soluble, bioactive signaling molecules^1^,^2^. *Drosophila* rhomboid proteases have been shown to act as the key activators of EGFR ligands^2^, but the function of the mammalian rhomboids is still ill defined. Here we show that the ER-resident rhomboid protease RHBDL4 regulates a non-canonical mechanism in response to GPCR signaling, which leads to microvesicular secretion of membrane-tethered proTGFα and other TM cargo^33^. Consistent with several previous reports^5^,^42^,^43^, we show that proTGFα is not directly cleaved by RHBDL4, although this contradicts a more recent report that RHBDL4-catalyzes proTGFα ectodomain shedding^16^. We do not have a full explanation for the discrepancy between our data and that reported by Song *et al*. Nevertheless, we note that we can detected traces of a 17-kDa species that corresponds to what they interpret to be the product of RHBDL4 cleavage, but that, in our experiments, this band was still present when a catalytic mutant of RHBDL4 was used, and with proTGFα mutants predicted to be uncleavable. This leads us to conclude that the minor 17-kDa species is not the product of RHBDL4 cleavage. We also note that in uncropped western blots shown in supplementary data, Song *et al*. also detected secretion of larger forms of TGFα. The mechanism we describe adds a new process by which rhomboid-family proteins influence the EGFR signaling pathway.

**Figure 7:**
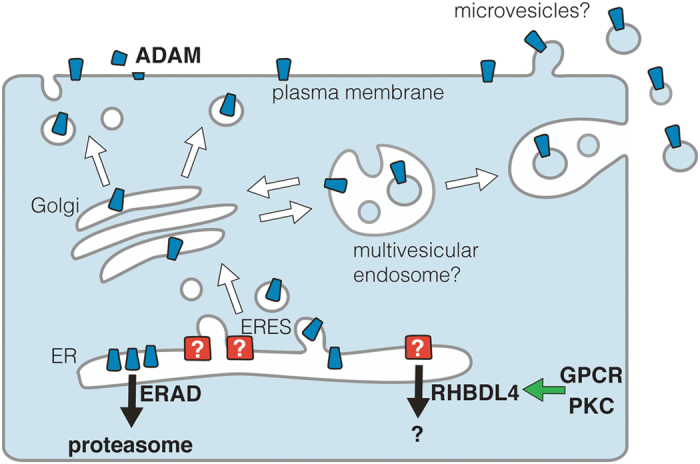
Model for RHBDL4 induced trafficking of proTGFα and secretion via microvesicles. Surplus proTGFα (indicated in blue) is constantly degraded by the ERAD pathway unless RHBDL4 removes an unknown ER-to-Golgi secretion block (indicated in red) probably by triggering degradation of a putative ER exit site (ERES) factor. Stimulating effect by GPCR and PKC that may increase RHBDL4 activity are highlighted by a green arrow.

ProTGFα is not significantly cleaved by RHBDL4, even when the protease is overexpressed, but instead it drives ER-to-Golgi transport of full-length membrane proTGFα and other cargoes by an unknown mechanism, which is dependent on RHBDL4 proteolytic activity. Given the ER localization of RHBDL4, we hypothesize that RHBDL4 might act on a factor involved in ER exit site regulation, hence explaining the ability to act on several TM cargoes (Fig. 7). The identity of this putative regulator, whether the RHBDL4-induced effect is mediated via regulatory degradation or proteolytic activation of this third party, and finally, the repertoire of TM cargoes affected, remain important unsolved questions. This model is consistent with our observation that the cytoplasmic tail of proTGFα is necessary and sufficient to promote RHBDL4 regulated ER-to-Golgi trafficking. The cytoplasmic tail of proTGFα has previously been shown to interact at various stages along the secretory pathway with regulatory factors including PDZ-domain proteins^11^.

Our results imply that ER-to-Golgi trafficking of proTGFα^16^ is rate-limiting and that the majority of ectopically expressed proTGFα does not leave the ER but instead is targeted for proteasomal degradation (Fig. 7) (this study and ref. 22). Although the physiological relevance of this mechanism needs to be evaluated, it is attractive to speculate that secretion of TGFα and other biologically important signaling molecules is controlled at the earliest stage in the secretory pathway: at the level of the ER exit site. This principle of signal regulation at the level of ER-Golgi trafficking has already been described. For example, the putative cargo receptor Star regulates ER exit of the *Drosophila* TGFα homologue Spitz^5^. Interestingly in that case, rhomboid proteases have been implicated in controlling the abundance of Star – a mechanism perhaps analogous to RHBDL4 control of proTGFα^44^. In another example, iRhoms, catalytically inactive relatives of rhomboid proteases, regulate the ER-to-Golgi trafficking of ADAM17^45^,^46^,^47^,^48^. Although these examples are distinct, a consistent principle emerges: like Star and iRhoms, RHBDL4 tunes the secretion dynamics of client proteins from within the early secretory pathway.

We show that RHBDL4-mediated secretion control can be modulated by GPCR signaling. This is consistent with growing body of evidence supporting the view that certain rhomboid-family proteins are regulated by cellular signaling^49^,^50^,^51^. We have previously shown that, in the context of ERAD, RHBDL4 specificity is governed by its cytoplasmic ubiquitin interacting motif, thereby linking recognition of unstable proteins to ER quality control and E3 ubiquitin ligases^17^. An important open question is whether there is a second regulatory mechanism of RHBDL4 activity or whether GPCR signaling modulates phosphorylation-dependent ubiquitination of putative RHBDL4 substrates as observed for the SCF E3 ubiquitin ligase complex during cell cycle^52^. There is clearly still much to understand mechanistically about how RHBDL4 controls trafficking.

It is now well recognized that EGFR transactivation acts as potent regulator of cell proliferation and cell invasion in many cell types including cancer cells, making it an attractive drug target^38^. The molecular mechanism, however, has not yet been fully resolved. Known molecular processes involved range from activation of ADAM-catalyzed shedding of growth factors^12^, to ligand-independent activation of receptor tyrosine kinases^38^, and iRhom mediated ER-Golgi trafficking of ADAM17^47^. RHBDL4 regulated trafficking provides a new mechanism by which GPCRs can boost biosynthesis and secretion of EGFR ligands.

Importantly, a GPCR-induced 37-kDa higher molecular weight form of TGFα has previously been observed in mice in the context of chronic kidney disease^14^, suggesting that the mechanism that we revealed may have pathological relevance. Furthermore, release of EGFR ligands by exosomes has been observed in human breast and colorectal cancer cells^33^. Whereas Coffey and co-workers showed that the EGFR ligand amphiregulin is continuously released in exosomes in a signaling-competent state, leading to increased cancer cell invasion, they showed, like us, that TGFα associated with the same vesicles fraction showed no significant activity^33^. Since membrane-anchored TGFα does not act as a juxtacrine EGFR ligand^53^, this explains why we and others did not detect any significant activity of the microvesicle-associated forms (this study and ref. 33). Instead, we observed that the 37-kDa species and 20-kDa TGFα-TMD form (lacking the pro-peptide; see Fig. 1A) can be trimmed to the bioactive 6-kDa species.

We do not know the functional consequence of microvesicle release of membrane tethered clients, including TGFα. Release of bioactive ligands may occur from extracellular microvesicles by shedding events analogous to the canonical processing along the secretory pathway. Consistent with this hypothesis, active sheddases have been described to be also released by exosomes and soluble proteases have been described that can release TGFα from its membrane anchor^32^,^54^,^55^. Alternatively, microvesicles may become internalized by target cells to inactivate the EGFR ligand by lysosomal degradation^33^. Serial action of regulated trafficking events with ADAM-type sheddases generating bioactive ligands may be in balance with catabolic processes such as regulatory ERAD and lysosomal degradation. Association of RHBDL4/*Rhbdd1* expression with colorectal cancer growth suggests that at least under pathogenic conditions this mechanism may promote EGFR signaling^16^.

Notably, we observed that the RHBDL4-mediated control of secretion dynamics is not restricted to proTGFα. CD44 is an important receptor for hyaluronic acid that mediates cell-to-cell and cell-to-matrix interaction, thereby controlling cell migration^56^. It is therefore conceivable that also CD44 levels need to be adapted to varying growth conditions. Likewise, changes in the CD44 expression level have been linked to numerous pathologies^56^. We do not know if there are further clients for the RHBDL4-controled secretion control, but as we hypothesize that RHBDL4 affects the cargo selection machinery, it seems likely. Overall, this work emphasizes the possibility that signaling pathways can regulate cargo export to buffer metabolic and signaling demands^36^. Our data also add weight to the importance of understanding the cell biological regulation of signaling pathways, and further highlights the central role of the rhomboid-like family in controlling the production of bioactive signals.

## Methods

### Plasmids

Unless otherwise stated proteins were cloned into pcDNA3.1+ (Invitrogen). Plasmids encoding mouse RHBDL1, RHBDL2, RHBDL3 and RHBDL4 tagged with an N-terminal triple HA-tag had been described previously^9^,^57^. Human proTGFα^5^ was used either untagged or tagged in the pro-peptide between residue 31 and 32 by a triple FLAG-tag (FLAG_3_). Rhomboid mutants and proTGFα-FLAG_3_ mutants were generated by Quick-Change site-directed mutagenesis (Stratagene). Deletion constructs proTGFαΔ4 and proTGFαΔ31 were generated by subcloning the respective region of the open reading frame. The construct proTGFα-CNX^TMD^ was generated by overlap extension PCR^58^, replacing amino acid 99 to 121 of proTGFα by residue 482 to 504 of human calnexin^17^. CNX-TGFα^tail^ was generated accordingly by replacing amino acids 505 to 592 of calnexin by residues 122 to 160 of proTGFα. The juxtamembrane poly-His-tag in proTGFα-H_8_ was introduced at position 94 of proTGFα. The open reading frames encoding human CD44 (IMAGE cDNA clone 4865289) and mouse S1P without pro-peptide (IMAGE cDNA clone 5310414) were cloned without their signal peptides into pcDNA3-based expression vector containing the Spitz signal peptide fused to a FLAG_3_-tag^9^. RHBDL4-GFP wt, the catalytic S144A mutant and the catalytic mutant with a defective UIM (S144A/L274A/L278A) were based on the monomeric variant of pEGFP-N1 (Clontech) as had been described previously^17^. Constructs for RFP-tagged RHBDL4 were generated by subcloning into pTagRFP-N (Evrogen). Constructs for mouse RHBDL2-GFP^57^, human pTα-FLAG_3_^17^, human prolactin^24^, *Drosophila* Spitz-FLAG_3_^24^, *Drosophila* Star^5^ and the ER marker RFP-KDEL^59^ were previously described. Mouse GRPR (IMAGE cDNA clone 40047100) was cloned untagged. The construct coding human Bcl-XL was a gift from Seamus Martin^60^.

### Cell Lines and Transfection

Hek293T, A431, and COS7 cells were grown in DMEM (Invitrogen), HCT116 cells in McCoy’s 5a medium (Invitrogen) supplemented with 10% fetal bovine serum at 37 °C in 5% CO_2_. Inducible stably transfected Hek293 T-Rex cells (Invitrogen) expressing an RHBDL4-specific shRNA were grown in presence of blasticidin (10 μg/ml) and G418 (0.5 mg/ml). Knockdown was induced with 1 μg/ml doxycycline. Transient transfections were performed using FuGENE 6 (Roche) or 25 kDa linear polyethylenimine (Polysciences)^61^ as described^5^,^62^. Typically, 250 ng plasmid encoding substrate/client and 25 ng plasmid encoding a rhomboid were used per well of a 6-well plate. Total transfected DNA (1 μg/well) was held constant by the addition of empty plasmid. For polyethylenimine transfection commonly double the amount of DNA was used. Sixteen hours post transfection, medium was replaced with serum-free Opti-MEM medium (Invitrogen) containing 10 µM BB94 (British Biotech) unless otherwise stated. For activation of endogenous rhomboid activity, 1 μM PMA (Sigma) or 100 nM bombesin (Sigma) was added to cell medium. For inhibitor studies, the indicated protease inhibitors (Calbiochem), diluted in DMSO, were compared with a carrier only. Medium was harvested typically after 24 to 30 hours; for inhibitor studies using DCI, a time course with 0 minutes, 30 minutes and 4 hour timepoints was performed. Cells were solubilized in SDS-sample buffer and analyzed by SDS-PAGE (see below). EndoH (New England Biolabs) and PNGaseF (New England Biolabs) treatment of SDS-solubilized cell extracts was performed according to the manufacturer’s instructions. Conditioned media were centrifuged for 5 minutes at 500 g followed by 10 minutes at full speed in a microfuge to remove cell debris, and subsequently proteins in the supernatant were precipitated by adding trichloroacetic acid (TCA) to 10%. The precipitate was recovered by centrifugation, washed with acetone and dissolved in SDS-PAGE sample buffer and analyzed by SDS-PAGE and western blotting (see below). Alternatively to TCA precipitation, proTGFα-H_8_ in conditioned medium was captured by metal-chelate chromatography using Ni-NTA agarose beads (Qiagen) in the presence of 20 mM imidazole at pH 8.0. Subsequently beads were washed with 20 mM Tris-Cl pH 8.0, 50 mM imidazole, eluted with SDS-sample buffer and analyzed by western blotting.

### EGFR Activation Assay

Subconfluent A431 cells were grown in serum free medium for 24 hours, followed by incubation with conditioned medium that had been harvested from a cellular RHBDL4-cleavage assay using untagged proTGFα (see above). After 10 minutes incubation at 37 °C, cells were lyzed in SDS-sample buffer and analyzed by western blotting (see below).

### Pulse-label Chase Analysis

For pulse-label chase experiments, transfected Hek293T cells were starved for 60 min in methionine/cysteine free DMEM (Invitrogen) supplemented with 10% dialysed fetal calf serum, then metabolically labelled for 10 min with 55 μCi/ml ^35^S-methionine/cysteine protein labeling mix (Perkin Elmer). Cells were rinsed with PBS, then chased in normal growth medium. At the end of the chase period cells were rinsed with PBS and solubilized in 1% Triton X-100 followed by immunoprecipitation of FLAG-tagged proteins as described below. Samples were analyzed by SDS-PAGE and labeled proteins were visualized by a FLA-7000 phosphorimager (Fuji).

### Immunoprecipitation

For substrate trapping, RHBDL4-GFP expressing Hek293T cells were solubilized with 1% Triton X-100 in IP buffer (50 mM HEPES-KOH, pH 7.4, 150 mM NaCl, 2 mM MgOAc_2_, 10% glycerol, 1 mM EGTA), containing EDTA-free complete protease inhibitor cocktail (Roche) and 10 μg/ml PMSF. Cell lysates were cleared by centrifugation at 20,000 g for 15 min and subsequently pre-incubated for 1 h on BSA-coupled sepharose beads. Anti-GFP immunoprecipitation was performed using a GFP-specific single chain antibody fragment coupled to NHS-activated sepharose beads as described^63^. Immunoprecipitates were washed three times in IP buffer, containing 0.1% Triton X-100 and eluted in SDS sample buffer and analyzed by western blotting (see below).

### Separation of Microvesicles by Continuous Sucrose Gradient Centrifugation

Microvesicles were isolated from conditioned medium by sucrose gradient centrifugation, as has been described^34^. All steps were performed at 4 °C. In brief, conditioned medium from 4 × 10 cm dishes of confluent cells was collected 40 h after transfection and was centrifuged at 400 g for 10 minutes, followed by 20,000 g for 20 minutes to remove cell debris. Subsequently, extracellular vesicles were pelleted by centrifugation at 175,000 g for 60 minutes and washed two times with PBS. The pellet was resuspended in 100 μl PBS and 1 ml of 2.5 M sucrose solution (dissolved in 25 mM HEPES-KOH, pH 7.2) was added. For the sucrose gradient, 6 ml of 2 M sucrose was overlaid with 6 ml of 0.25 M sucrose in a polycarbonate centrifuge tube and mixed using a Gradient Master (BioComp Instruments). The sucrose gradient was underlaid with the dissolved vesicle-containing pellet and centrifuged using a SW40 Ti swinging bucket rotor at 100,000 g for 16 h. Subsequently, nine 1.5 ml fractions were collected and precipitated with 20% TCA and analyzed by western blotting (see below).

### Antibodies

The following antibodies were used: mouse monoclonal anti-FLAG (M2; Sigma), mouse monoclonal anti-HA (HA.11; Covance), mouse monoclonal anti-TGFα (134A-2B3; Oncogene), mouse monoclonal anti-phospho-EGFR antibody (9H2; Upstate), polyclonal rabbit anti-EGFR (1005; Santa Cruz Biotechnology), polyclonal rabbit anti-Prl (Serotec), mouse monoclonal anti-BAP31 (A1/182; Alexis). Polyclonal rabbit anti-GFP antibody was a gift from Dirk Görlich. Polyclonal rabbit antibody specific for RHBDL4 has been described previously^17^. For affinity purification the GST fusion protein was coupled to HiTrap NHS-activated HP (Amersham Biosciences) and used to purify the antibody according to standard protocols.

### Microscopy

For life cell analysis, HCT116 cells were analyzed by confocal microscopy using a LSM 780 confocal microscope (Zeiss). For immunofluorescence analysis, COS7 cells were fixed in methanol at −20 °C for 5 minutes followed by acetone at −20 °C for 45 seconds. Cell were washed with PBS and blocked with 20% fetal calf serum in PBS, cells were probed with affinity-purified anti RHBDL4 antibody (1:500; see above) and anti BAP31 antibody (1:1000). After staining with fluorescently labeled secondary antibody (Santa Cruz Biotechnology), slides were analyzed using the LSM 780 confocal microscope.

### Western Blotting

Transfected cells, TCA-precipitated tissue culture supernatants and fractions from sucrose gradient centrifugation were solubilized in SDS sample buffer (50 mM Tris-Cl pH 6.8; 10 mM EDTA, 5% glycerol, 2% SDS, 0.01% bromphenol blue) containing 5% β-mercaptoethanol. All samples were incubated for 15 min at 65 °C, resolved on Tris-glycine SDS-PAGE followed by western blot analysis using enhanced chemiluminescence to detect bound antibodies. In order to detect the 6-kDa form of TGFα, proteins were transferred on PVDF membrane with 0.2 μm pore size (Millipore) and probed with TGFα-specific antibody (1:100). For detection of phosphorylated EGFR, PVDF membranes were blocked in 3% BSA in TBS-Tween supplemented with 200 μM NaVO_3_ and probed with anti phospho-EGFR antibody (1:2000). Subsequently membranes were stripped and reprobed with the EGFR-specific antibody (1:1000). For detection, X-ray films or the LAS-4000 system (Fuji) were used.

## Additional Information

**How to cite this article**: Wunderle, L. *et al*. Rhomboid intramembrane protease RHBDL4 triggers ER-export and non-canonical secretion of membrane-anchored TGFα. *Sci. Rep.* **6**, 27342; doi: 10.1038/srep27342 (2016).
